# Roux-en-Y Jejunal Limb Obstruction without Intrahepatic Bile Duct Dilatation after Kasai Portoenterostomy in an Adult with Biliary Atresia: A Case Report

**DOI:** 10.70352/scrj.cr.26-0313

**Published:** 2026-11-06

**Authors:** Shoichi Tsuzaka, Kyoichi Deie, Motoki Ebihara, Rina Matsuda, Shoko Ogawa, Shotaro Taki, Itsuki Naya, Yoshihiro Hirata, Koichi Mizuta, Takahiro Hosokawa, Hiroshi Kawashima

**Affiliations:** 1 Department of Pediatric Surgery, Saitama Children’s Medical Center, Saitama, Saitama, Japan; 2 Department of Transplant Surgery, Saitama Children’s Medical Center, Saitama, Saitama, Japan; 3 Department of Radiology, Saitama Children’s Medical Center, Saitama, Saitama, Japan

**Keywords:** biliary atresia, Kasai portoenterostomy, Roux-en-Y jejunal limb, hepatobiliary scintigraphy, obstructive jaundice

## Abstract

**INTRODUCTION:**

Biliary atresia (BA) is typically managed with Kasai portoenterostomy (KPE). While long-term complications, such as cholangitis and portal hypertension, are well known, mechanical obstruction of the Roux-en-Y jejunal limb is rare following KPE. Diagnosis of such obstruction can be challenging when conventional imaging fails to show dilatation of the intrahepatic bile ducts or Roux-en-Y limb.

**CASE PRESENTATION:**

A 22-year-old man with BA, who had undergone KPE at 50 days of age, presented with obstructive jaundice and fever. Although CT and magnetic resonance cholangiopancreatography showed no intrahepatic biliary dilatation, CT suggested extrinsic compression of the Roux-en-Y limb by a dilated duodenum. Technetium-99m N-pyridoxyl-5-methyl-tryptophan hepatobiliary scintigraphy (HBS) demonstrated impaired bile flow, with tracer retention within the Roux-en-Y limb and a markedly reduced 60-min excretion rate of 8.2%. Open adhesiolysis was performed, revealing that the Roux-en-Y limb was compressed by adhesions to the duodenum and surrounding organs. Postoperative recovery was uneventful, with immediate improvement in liver function. Follow-up HBS performed 3 months after surgery showed markedly improved bile drainage, with the 60-min excretion rate increasing to 58.8%.

**CONCLUSIONS:**

This case illustrates that mechanical bile outflow obstruction can occur without intrahepatic bile duct dilatation in adult patients with BA after KPE. HBS is a useful functional imaging modality for assessing dynamic bile flow and should be considered when clinical suspicion of obstruction remains despite inconclusive conventional imaging.

## Abbreviations

BA
: biliary atresia
HBS
: hepatobiliary scintigraphy
KPE
: Kasai portoenterostomy
MRCP
: magnetic resonance cholangiopancreatography
Tc-99m PMT
: technetium-99m N-pyridoxyl-5-methyl-tryptophan

## INTRODUCTION

BA is a progressive fibro-obliterative cholangiopathy of infancy that involves the intrahepatic and extrahepatic bile ducts, resulting in cholestasis and progressive liver failure. Without surgical intervention, this condition is uniformly fatal within the first years of life.^1–3)^ A major advance in the management of BA was the development of KPE. In KPE, the fibrotic extrahepatic biliary remnant is excised, and a Roux-en-Y jejunal limb is anastomosed directly to the porta hepatis to restore bile drainage.^1)^

Despite these advances, long-term complications, such as recurrent cholangitis, progressive fibrosis, portal hypertension, and the eventual need for liver transplantation, remain common after KPE.^1,4,5)^ Complications of the Roux-en-Y jejunal limb have been frequently reported after gastrectomy for gastric cancer and pancreaticoduodenectomy for pancreatic head cancer. These complications include inflow tract obstruction due to adhesions, torsion, or internal hernia, which can result in acute abdomen or obstructive jaundice.^6,7)^ Conversely, complications attributable to the Roux-en-Y jejunal limb after KPE are rare.

Roux-en-Y jejunal limb obstruction is typically diagnosed using ultrasonography or CT, which may demonstrate the dilatation of intrahepatic bile ducts or the Roux-en-Y jejunal limb. However, BA affects the intrahepatic biliary tree as well as the extrahepatic bile ducts, and progressive fibro-obliterative changes may render the intrahepatic ducts less prone to dilatation in response to downstream obstruction. Furthermore, in cases of partial narrowing rather than complete obstruction, bile outflow is impaired without obvious dilatation of the Roux-en-Y limb. Therefore, mechanical bile outflow obstruction may be difficult to diagnose using morphological imaging alone.^3,6–12)^

By contrast, HBS provides a dynamic functional assessment of bile excretion and enteric flow and has been widely used in the evaluation of BA before and after surgery.^13–16)^ However, to our knowledge, reports describing the use of HBS to identify Roux-en-Y jejunal limb complications after KPE are extremely limited. We describe a rare case of bile outflow obstruction due to extrinsic compression of the Roux-en-Y limb following KPE, in which HBS demonstrated impaired bile drainage and contributed to the decision to perform surgical adhesiolysis.

## CASE PRESENTATION

A 22-year-old man with BA who had undergone KPE at 50 days of age presented with jaundice and fever. After reaching adulthood, he experienced 1 episode of cholangitis accompanied by jaundice and elevated liver enzymes. Aside from this episode, he had maintained a generally stable postoperative course under regular follow-up at our institution, without overt jaundice or clinically apparent complications of portal hypertension; however, total bilirubin had remained mildly elevated (approximately 1 mg/dL) from approximately 13 years and 8 months after KPE (POD 5000) onward (**Fig. 1A**).

**Fig. 1:**
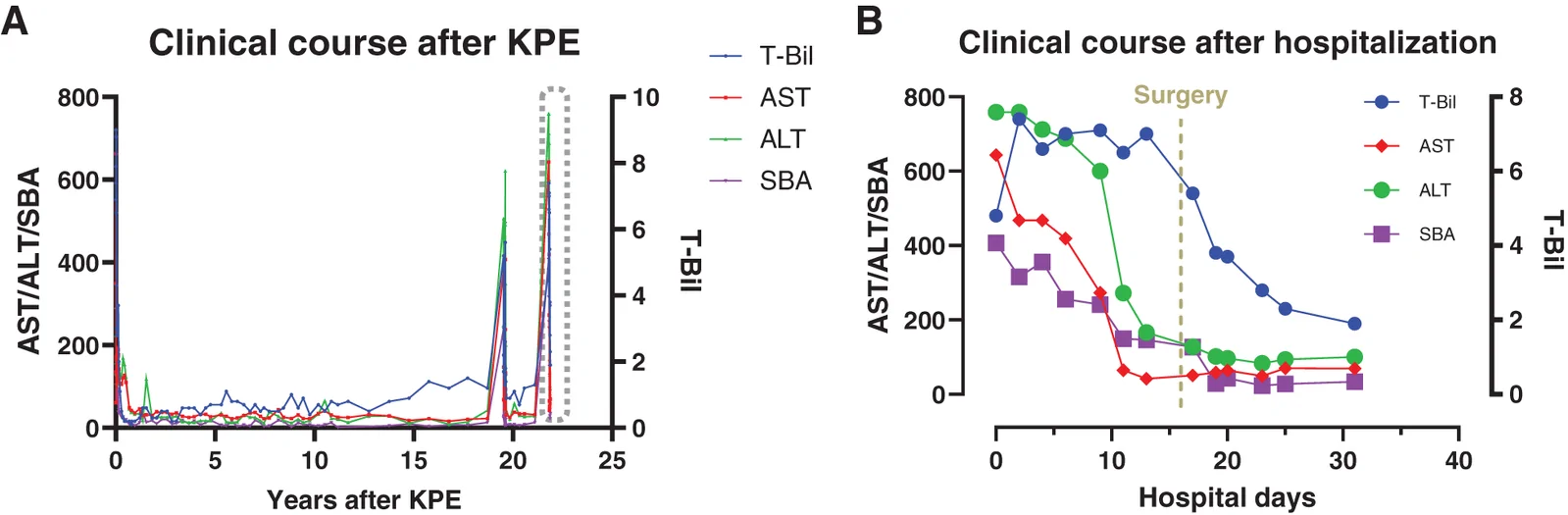
Clinical courses. (**A**) Clinical course of liver function after KPE. Apart from 1 episode of cholangitis after reaching adulthood, the patient remained clinically stable with his native liver. The gray dotted rectangle indicates the period shown in panel (**B**). (**B**) Clinical course after the present admission. Serum bilirubin, liver enzyme, and serum bile acid levels decreased promptly after surgery. The brown dotted line indicates the day of surgery. ALT, alanine aminotransferase; AST, aspartate aminotransferase; KPE, Kasai portoenterostomy; SBA, serum bile acids; T-Bil, total bilirubin

On admission (approximately 21 years and 9 months after KPE [POD 7964]), laboratory tests showed hyperbilirubinemia (total bilirubin: 4.8 mg/dL, direct bilirubin: 2.8 mg/dL), markedly elevated liver enzyme levels (aspartate aminotransferase: 643 U/L, alanine aminotransferase: 759 U/L), and an elevated serum bile acid level (407.0 µmol/L; **Table 1**). He was hospitalized with a presumptive diagnosis of cholangitis and treated with antibiotics. Abdominal CT and MRCP showed no intrahepatic bile duct dilatation or obstructive lesions (**Fig. 2**). CT also revealed splenomegaly, with the spleen measuring 9.65 × 4.66 × 12.46 cm, but no definite splenorenal shunt or gastrointestinal varices. The platelet count was mildly decreased at 15.0 × 10^4^/µL (reference range, 16.1–33.7 × 10^4^/µL), suggesting mild hypersplenism without other overt manifestations of portal hypertension.

**Table 1:** Laboratory findings on admission

Complete blood count		Normal reference ranges
White blood cell count	5450/μL	3700–8200
Hemoglobin	16.9 g/dL	13.6–16.3
Platelet count	15.0 × 10^4^/μL	16.1–33.7 × 10^4^
Prothrombin time	12.1 s	9–13
Activated partial thromboplastin time	24.5 s	23.0–36.0
Biochemical tests		
Total protein	8.6 g/dL	6.3–8.3
Albumin	4.8 g/dL	3.8–5.3
Total bilirubin	4.8 mg/dL	0.2–1.4
Direct bilirubin	2.8 mg/dL	0.0–0.3
Aspartate aminotransferase	643 U/L	10–35
Alanine aminotransferase	759 U/L	5–45
Alkaline phosphatase	1190 U/L	35–120
Amylase	60 IU/L	37–132
Blood urea nitrogen	8.0 mg/dL	7–22
Creatinine	0.7 mg/dL	0.6–1.2
Sodium	139 mEq/L	135–145
Potassium	3.5 mEq/L	3.4–5.0
C-reactive protein	0.1 mg/dL	≤0.3
Serum bile acid	407.0 µmol/L	≤10
Mac-2-binding protein glycosylation isomer	0.8 AU/mL	<1.0

**Fig. 2:**
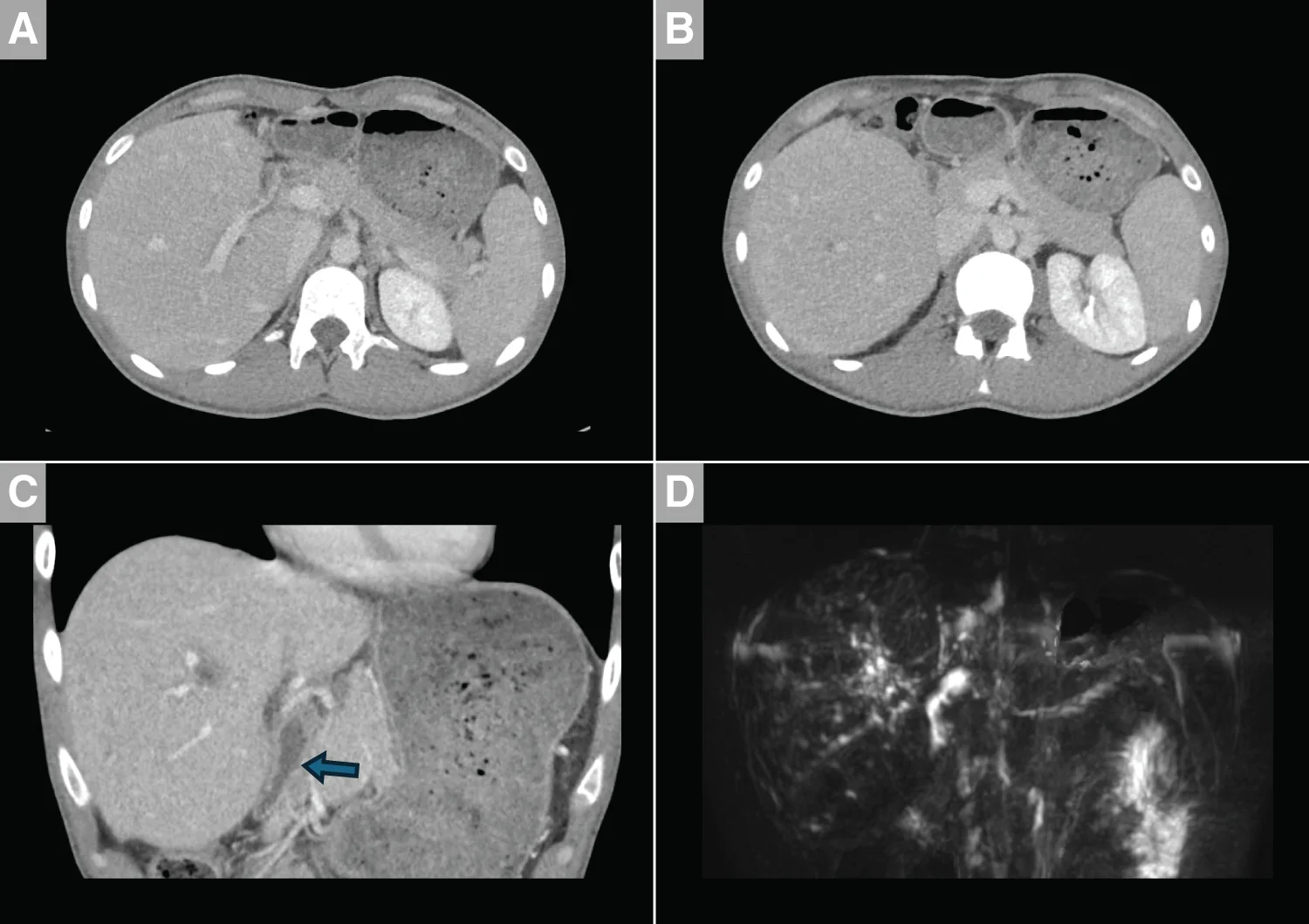
CT and MRCP images. (**A**, **B**) Axial CT images. Panel (**A**) shows a more cranial slice. No intrahepatic bile duct dilatation is observed. Splenomegaly is also present, without a definite splenorenal shunt or gastrointestinal varices. (**C**) Coronal CT image. The arrow indicates a dilated duodenum. No dilatation of the intrahepatic bile ducts is observed. (**D**) MRCP image. No dilatation of the intrahepatic bile ducts is observed. MRCP, magnetic resonance cholangiopancreatography

CT also showed that the dilated duodenum appeared to compress the Roux-en-Y limb, possibly in association with postoperative adhesions. Tc-99m PMT HBS revealed tracer retention within the Roux-en-Y limb, with a markedly reduced 60-min excretion rate of 8.2%. These findings indicated impaired bile drainage and supported the diagnosis of mechanical bile outflow obstruction involving the Roux-en-Y limb (**Fig. 3**). Although CT showed dilation of the stomach and duodenum, the patient had no obstructive symptoms, such as vomiting, even after oral intake; this indicated that gastrointestinal passage through the stomach and duodenum was preserved. The cause of the duodenal dilatation remained uncertain but may have been related to inflammatory changes associated with cholangitis.

**Fig. 3:**
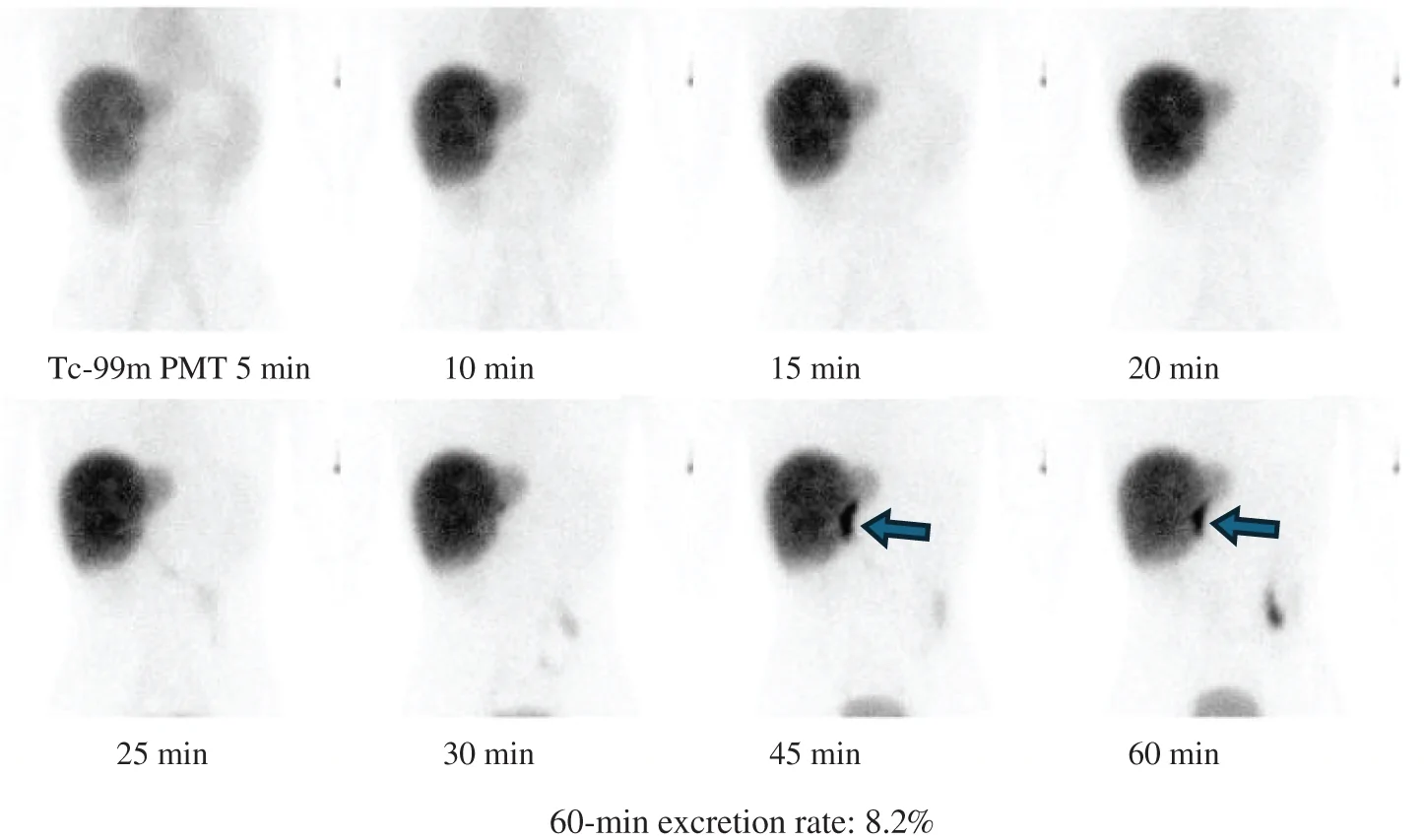
Tc-99m PMT HBS. Tracer retention within the Roux-en-Y limb is observed beginning 45 min after injection and persisting through 60 min; the 60-min excretion rate was 8.2%, indicating markedly delayed biliary excretion. The arrows indicate the site of tracer retention. HBS, hepatobiliary scintigraphy; Tc-99m PMT, technetium-99m N-pyridoxyl-5-methyl-tryptophan

Open adhesiolysis was performed on hospital day 16. The Roux-en-Y limb was adherent to the duodenum, stomach, omentum, and transverse colon. These intraoperative findings were consistent with, but more extensive than, the preoperative hypothesis of isolated duodenal compression, as the stomach, omentum, and transverse colon also contributed to the extrinsic compression (**Fig. 4A**). After careful adhesiolysis up to the Roux-en-Y anastomosis, the Roux-en-Y limb straightened and the compression was relieved. The operative time was 102 min, and the blood loss was 8 mL (**Fig. 4B**). No intrinsic stricture or other intraluminal obstruction was identified; therefore, neither resection nor reconstruction of the Roux-en-Y limb was required.

**Fig. 4:**
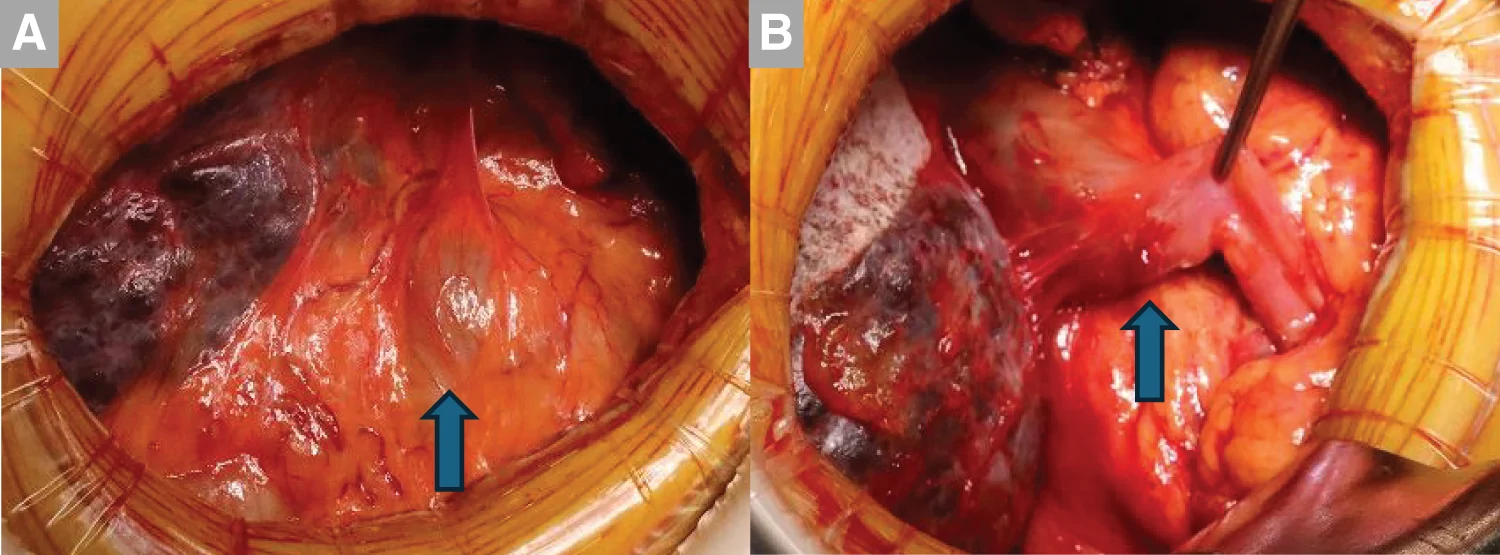
Intraoperative findings. (**A**) Immediately after laparotomy, the Roux-en-Y limb was not visible because it was covered by the greater omentum and transverse colon. The Roux-en-Y limb was adherent to the duodenum, omentum, stomach, and transverse colon. The arrow indicates the transverse colon. (**B**) After adhesiolysis, the Roux-en-Y limb was exposed. The arrow indicates the Roux-en-Y limb.

Postoperatively, serum bilirubin and liver enzyme levels began to improve from POD 1, and the patient was discharged on POD 9 (**Fig. 1B**). Follow-up Tc-99m PMT HBS 3 months after surgery showed marked improvement in bile drainage, with the 60-min excretion rate increasing from 8.2% to 58.8% (**Fig. 5**). The patient remained well without recurrence during subsequent follow-up.

**Fig. 5:**
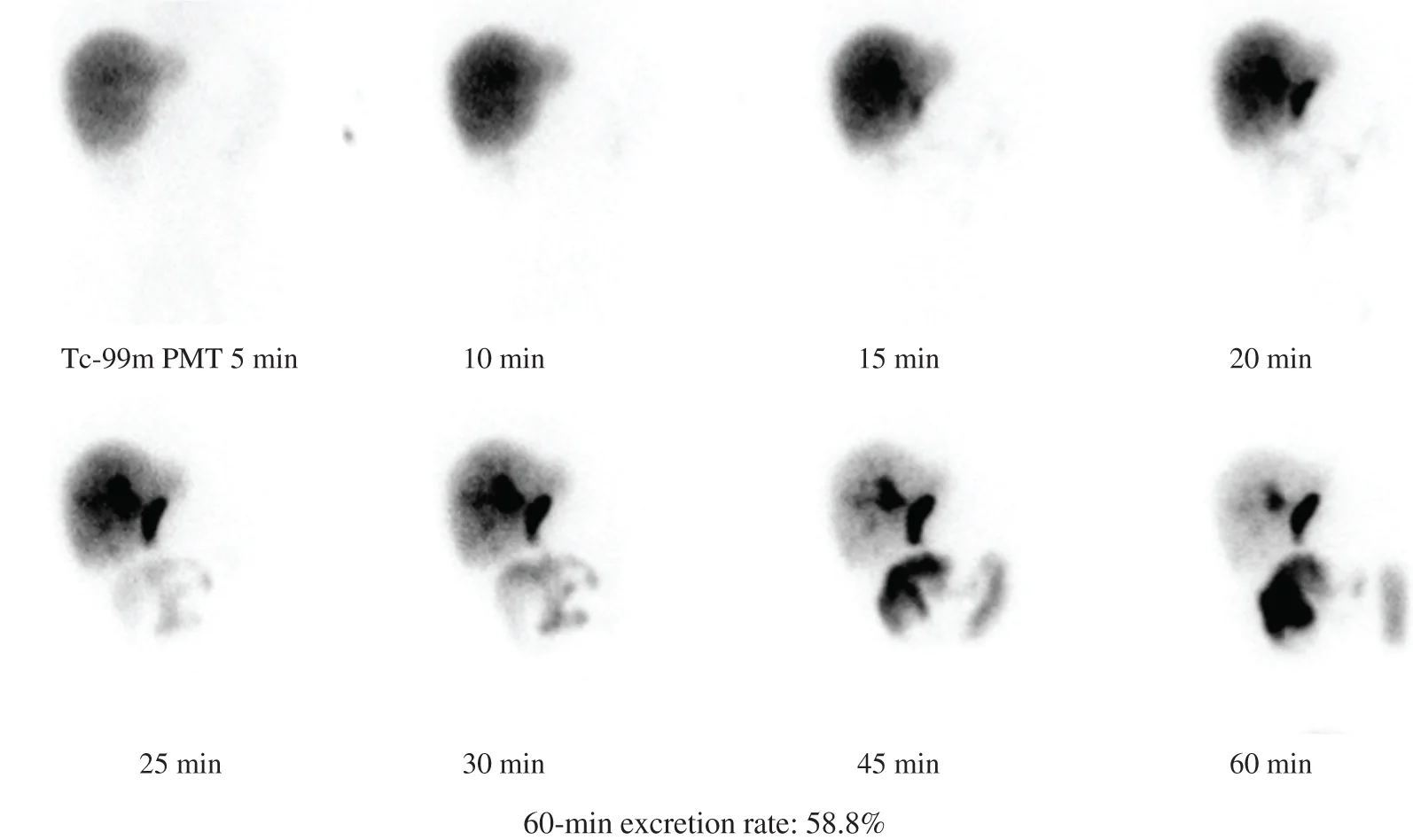
Follow-up Tc-99m PMT HBS performed 3 months after surgery. Compared with the preoperative findings, the tracer passage into the distal bowel is markedly improved, with the 60-min excretion rate increasing from 8.2% to 58.8%. HBS, hepatobiliary scintigraphy; Tc-99m PMT, technetium-99m N-pyridoxyl-5-methyl-tryptophan

## DISCUSSION

This case represents a rare instance of bile outflow obstruction caused by extrinsic compression of the Roux-en-Y limb after KPE. Sixteen days elapsed between admission and surgery, mainly because CT and MRCP showed no dilatation of the intrahepatic bile ducts or the Roux-en-Y limb, leading us to initially underestimate the possibility of mechanical obstruction. However, HBS clearly demonstrated localized tracer retention within the Roux-en-Y limb, together with markedly delayed biliary excretion. HBS was particularly useful because it allowed functional assessment of bile excretion even in the absence of obvious morphological abnormalities on CT or MRCP.

BA involves not only the extrahepatic but also the intrahepatic bile ducts.^10)^ The intrahepatic bile ducts in BA undergo a progressive fibro-obliterative process characterized by periductal and portal fibrosis, ductular reaction, narrowing, and progressive obliteration of the ductal lumen.^11,12)^ A histopathological study revealed intrahepatic bile duct obstruction in 42.9% and bile duct paucity in 20.0% of patients with BA, with bile ducts significantly narrower than those in healthy children.^3)^ These intrinsic structural changes may reduce the caliber and compliance of the intrahepatic bile ducts, making them less likely to dilate in response to downstream bile outflow obstruction than previously normal bile ducts. This pathological feature may partly explain why CT and MRCP failed to demonstrate intrahepatic bile duct dilatation in the present case despite mechanical obstruction of the Roux-en-Y limb. Therefore, the absence of intrahepatic bile duct dilatation on morphological imaging does not exclude mechanical bile outflow obstruction in patients with BA after KPE.

Tc-99m PMT HBS allows for the dynamic assessment of bile excretion and passage into the intestine. In previous pediatric liver transplantation studies, HBS has been used to evaluate biliary obstruction and stricture.^17,18)^ In one of these studies, the mean 60-min excretion rate was 78.0 ± 9.7% in patients without biliary stricture, and a cutoff value of 69.2% was proposed for detecting biliary stricture.^17)^ However, these values were derived from pediatric liver transplant recipients and should not be directly regarded as diagnostic thresholds for late mechanical obstruction of the Roux-en-Y limb after KPE. In our case, the 60-min excretion rate was markedly reduced to 8.2%, and focal tracer retention was observed within the Roux-en-Y limb. These findings provided functional evidence of impaired bile drainage and, together with the CT findings and clinical course, supported the diagnosis of mechanical bile outflow obstruction involving the Roux-en-Y limb.

Delayed tracer excretion on HBS alone is not specific for mechanical obstruction because impaired hepatocellular function and intrahepatic cholestasis may also delay biliary excretion. Therefore, HBS findings should be interpreted together with the distribution of tracer retention, morphological imaging findings, laboratory data, clinical symptoms, and response to medical treatment. In the present case, localized tracer retention within the Roux-en-Y limb, rather than diffuse hepatic retention alone, was particularly suggestive of impaired passage through the reconstructed jejunal limb. Follow-up HBS performed 3 months after adhesiolysis showed a marked increase in the 60-min excretion rate from 8.2% to 58.8% and improved tracer passage into the distal bowel. Although the postoperative excretion rate remained lower than the values reported in pediatric liver transplant recipients without biliary stricture, the marked improvement after relief of the compression further supported the diagnosis of mechanical bile outflow obstruction.

Beyond its use in the immediate postoperative period, HBS has also been shown to have prognostic value at later time points after KPE. The hepatic extraction fraction, excretion half-life, shape of the excretion curve, and Roux loop tracer activity on HBS performed at 1 week and 6 months after KPE have each been correlated with medium-term outcome.^15)^ Furthermore, free tracer excretion together with a serum bilirubin concentration below 57 µmol/L at 6 weeks after KPE have been identified as favorable prognostic indicators.^16)^ Although these previous studies primarily evaluated postoperative prognosis rather than late mechanical obstruction, they support the value of HBS for evaluating bile excretion and passage through the Roux-en-Y limb.

In patients presenting with jaundice or cholangitis long after KPE, prompt passage of tracer into the distal bowel without focal retention within the Roux-en-Y limb would argue against clinically significant mechanical obstruction and may support continued medical management and close observation, particularly when symptoms and laboratory abnormalities improve with conservative treatment. Conversely, persistent or localized tracer retention within the Roux-en-Y limb, markedly delayed excretion, progressive biochemical abnormalities, or a poor response to medical treatment should raise suspicion of a mechanical cause and prompt further evaluation. Nevertheless, HBS findings alone should not be regarded as an absolute indication for surgery; the decision to undertake surgical exploration should be based on the combined clinical, biochemical, morphological, and functional findings.

Based on this experience, we propose the following diagnostic approach for patients with BA presenting with jaundice or cholangitis long after KPE. CT, ultrasonography, and/or MRCP should first be performed to evaluate the intrahepatic bile ducts, Roux-en-Y limb, intra-abdominal collections, and other structural abnormalities. If these studies are inconclusive but clinical suspicion of impaired bile drainage persists, HBS should be considered to evaluate bile excretion dynamically. If HBS demonstrates focal tracer retention or delayed passage through the Roux-en-Y limb, the possibility of mechanical obstruction should be reassessed, even in the absence of intrahepatic bile duct dilatation. Surgical or other interventional treatment may then be considered according to the severity and persistence of the symptoms, laboratory abnormalities, response to conservative treatment, and overall imaging findings. In contrast, preserved tracer passage together with clinical and biochemical improvement would support continued conservative management and observation.

Although complications arising from the Roux-en-Y or afferent jejunal limb have been reported after other procedures, we found no previous reports specifically describing extrinsic compression of the Roux-en-Y limb after KPE. A comparable diagnostic scenario has, however, been described following other types of gastrointestinal or biliary reconstruction. Recurrent cholangitis due to nonobstructive afferent loop biliary stasis after pylorus-preserving pancreatoduodenectomy was clarified by HBS demonstrating tracer retention within the afferent jejunal limb.^6)^ Delayed excretion and localized tracer accumulation have also been reported in a liver transplant recipient with obstruction of a Roux-en-Y hepaticojejunostomy.^19)^ These reports support the use of functional imaging when morphological imaging does not adequately explain cholangitis or bile stasis in patients with surgically reconstructed jejunal limbs.

The apparent rarity of Roux-en-Y limb-related complications after KPE does not necessarily mean that such complications are truly uncommon. When Roux-en-Y limb dysfunction causes jaundice or cholangitis without intrahepatic bile duct dilatation, the condition may be attributed to recurrent cholangitis or progressive hepatic dysfunction associated with BA, and the underlying mechanical cause may remain unrecognized. In our patient, the obstruction was caused by extrinsic compression resulting from adhesions to the duodenum, stomach, omentum, and transverse colon, without kinking, torsion, intrinsic stricture, or intraluminal obstruction of the Roux-en-Y limb. Therefore, neither revision of the Roux-en-Y reconstruction nor resection of the limb was required, and adhesiolysis alone was sufficient to relieve the compression.

As increasing numbers of patients with BA survive into adulthood with their native livers, such late complications related to previous reconstruction may become increasingly relevant during long-term follow-up. Mechanical dysfunction of the Roux-en-Y limb should therefore be included in the differential diagnosis when a patient presents with jaundice or cholangitis after KPE, even when conventional imaging shows no intrahepatic bile duct dilatation. In such cases, HBS can complement morphological imaging by providing functional information regarding bile drainage and the site of tracer retention.

In summary, patients with BA may develop mechanical bile outflow obstruction without intrahepatic bile duct dilatation. Therefore, the absence of bile duct dilatation on CT or MRCP should not be considered sufficient to exclude Roux-en-Y limb dysfunction. HBS is a useful adjunct to morphological imaging for assessing bile drainage and may contribute to the diagnosis and treatment planning when conventional imaging findings are inconclusive.

## CONCLUSIONS

This case highlights the diagnostic and clinical value of HBS in identifying mechanical bile outflow obstruction caused by compression of the Roux-en-Y limb after KPE. When patients with BA present with obstructive jaundice after KPE, Roux-en-Y limb dysfunction should be included in the differential diagnosis even if intrahepatic bile duct dilatation is absent on imaging. HBS serves as a useful adjunct to morphological imaging by providing functional information regarding bile drainage and may aid in diagnosis and treatment planning in such patients.
